# Nitroso-Oxidative Stress, Acute Phase Response, and Cytogenetic Damage in Wistar Rats Treated with Adrenaline

**DOI:** 10.1155/2018/1805354

**Published:** 2018-11-21

**Authors:** Milena Radaković, Sunčica Borozan, Ninoslav Djelić, Saša Ivanović, Dejana Ćupić Miladinović, Marko Ristanić, Biljana Spremo-Potparević, Zoran Stanimirović

**Affiliations:** ^1^Department of Biology, Faculty of Veterinary Medicine, University of Belgrade, Bulevar oslobodjenja 18, 11000 Belgrade, Serbia; ^2^Department of Chemistry, Faculty of Veterinary Medicine, University of Belgrade, Bulevar oslobodjenja 18, 11000 Belgrade, Serbia; ^3^Department of Pharmacology and Toxicology, Faculty of Veterinary Medicine, University of Belgrade, Bulevar oslobodjenja 18, 11000 Belgrade, Serbia; ^4^Department of Physiology, Faculty of Pharmacy, University of Belgrade, Vojvode Stepe 450, 1221 Belgrade, Serbia

## Abstract

This study is aimed at analysing biochemical and genetic endpoints of toxic effects after administration of adrenaline. For this purpose, the study was carried out on Wistar rats and three doses of adrenaline were used: 0.75 mg/kg, 1.5 mg/kg, and 3 mg/kg body weight. To achieve these aims, we investigated the effects of adrenaline on catalase (CAT), Cu, Zn-superoxide dismutase (SOD), malondialdehyde (MDA), nitrite (NO_2_−), carbonyl groups (PCC), and nitrotyrosine (3-NT). Total activity of lactate dehydrogenase (LDH), its relative distribution (LDH_1_–LDH_5_) activity, level of acute phase proteins (APPs), and genotoxic effect were also evaluated. The obtained results revealed that all doses of adrenaline induced a significant rise in CAT activity, MDA level, PCC, NO_2_^−^, and 3-NT and a significant decrease in SOD activity compared to control. Adrenaline exerted an increase in total activity of LDH, LDH_1_, and LDH_2_ isoenzymes. Further study showed that adrenaline significantly decreased serum albumin level and albumin-globulin ratio, while the level of APPs (*α*_1_-acid glycoprotein and haptoglobulin) is increased. The micronucleus test revealed a genotoxic effect of adrenaline at higher concentrations (1.5 mg/kg and 3 mg/kg body weight) compared to untreated rats. It can be concluded that adrenaline exerts oxidative and nitrative stress in rats, increased damage to lipids and proteins, and damage of cardiomyocytes and cytogenetic damage. Obtained results may contribute to better understanding of the toxicity of adrenaline with aims to preventing its harmful effects.

## 1. Introduction

Adrenaline (epinephrine) is a naturally occurring catecholamine which is secreted by the medulla of the adrenal glands. As a drug, adrenaline was discovered over a century ago and has been used in human cardiopulmonary resuscitation since 1922 [1]. Adrenaline also finds application in treatment of cardiac arrest, asthma, allergic reactions, and glaucoma [2]. Adrenaline acts through the alpha and beta adrenergic receptors which leads to vasoconstriction, an increase in the rate and force of contraction of the heart, and dilatation of bronchi and cerebral vessels. During normal physiological conditions, there is no constant secretion of adrenaline, but under the stress condition, a high level of adrenaline is released to prepare body for “fight or flight” response [3].

However, adrenaline at other catecholamine at doses exceeding physiological levels may cause toxic effects [4, 5]. There are studies indicating toxic effects of adrenaline via signal transduction pathways [6, 7]. Also, it seems that adrenaline exerts detrimental effects via oxidative products of its metabolism [8–10]. In line with this, it was shown that autooxidation of catecholamine may generate reactive oxygen species (ROS) [11]. One of the final products of oxidative metabolism-adrenochrome is able to stimulate oxidation of adrenaline in which superoxide anion (O_2_^·−^) and hydrogen peroxide (H_2_O_2_) are formed. Besides, it was reported that catalase and quercetin diminished the DNA-damaging effect of adrenaline *in vitro* [12].

It is well known that overproduction of ROS can lead to disruption of redox balance and cause oxidative stress [13]. Namely, if antioxidative mechanisms fail to remove excess level of ROS, cells become prone to damage of DNA, proteins, and lipids [14]. Proteins are among major targets of ROS or reactive nitrogen species (RNS) and lead to changes in protein content such as carbonyl group formation and nitrotyrosine (3-NT) generation [15]. There are assumptions that high level of adrenaline may cause protein damage via free radicals [16]. It is worth noting that oxidative damage to proteins plays an important role in loss of physiological functions, thus favoring development of various diseases [17–19]. On the other hand, lipid peroxidation by ROS may initiate the peroxidation of membrane lipids and cause cellular injuries. In addition, oxidative DNA damages play a role in the mutagenesis and an increased risk of tumors [20, 21].

Although there are indications that adrenaline could increase the level of ROS, the cause-consequence relationship between them is not fully understood. For this purpose, we determined parameters of oxidative status: catalase (CAT) Cu, Zn-superoxide dismutase (SOD), malondialdehyde (MDA), nitrite (NO_2_^−^), lactate dehydrogenase (LDH), and carbonyl groups (PCC) after administration of adrenaline in Wistar rats. Also, we evaluated how adrenaline influenced the level of acute phase proteins (APPs), *α*_1_-acid glycoprotein (AGP), haptoglobin (Hp), and level of 3-NT. Possible genotoxic effects of adrenaline on bone marrow cells using micronucleus test were also estimated. The results of this investigation should contribute to a better understanding of adrenaline toxicity with aims to preventing its harmful effects.

## 2. Materials and Methods

### 2.1. Animals

In this study, Wistar rats aged 18 weeks weighing 220–280 g were obtained from the Institute of Biomedical Research, University of Belgrade. Three experimental groups with adrenaline and two control groups (negative and positive) contained seven male Wistar rats. The animals were kept under controlled constant environmental conditions (25 ± 4°C, 55 ± 5% humidity) with a 12/12 h light/dark cycle. They received food and water *ad libitum*. Care and animal treatment were conducted according to the Guide for the Care and Use of Laboratory Animals (National Research Council [22]). The investigation was approved by the Ethical Committee of the Faculty of Veterinary Medicine (University of Belgrade).

### 2.2. Doses and Treatment

Three experimental doses of adrenaline were used: 0.75 mg/kg, 1.5 mg/kg, and 3 mg/kg body weight, corresponding to 15%, 30%, and 60%, respectively, of LD50 [23]. Cyclophosphamide was used as a positive control and 60 mg/kg body weight per rats was given, while the negative control group was treated with saline (0.9% NaCl) [24]. The intraperitoneal (i.p.) route of application was used in all experimental groups. After 24 hours of treatments, all rats were euthanized. From each animal, blood and bone marrow from both femurs were taken.

### 2.3. Blood Sampling

Blood samples for the biochemical analysis were taken from the rats by puncture of *v. jugularis* and collected into heparinized tubes. Plasma was obtained from blood by centrifugation at 3000 rpm for 10 min. Erythrocytes were rinsed three times in isotonic solution of NaCl (0.9%). Then, samples were frozen at −20°C until analysis. Haemoglobin concentration was estimated by the cyanomethaemoglobin method [25]. Haemolysates of erythrocytes were used for determination of activities of antioxidant enzymes (SOD, CAT) and level of MDA. Plasma was used for determination of NO_2_^−^ concentration, 3-NT, protein carbonyl groups, total LDH level and its isoenzyme activity, and levels of APPs, Hp, and AGP.

### 2.4. Oxidative Stress Parameters

The Cu, Zn-superoxide dismutase (SOD) activity in erythrocytes was determined spectrophotometrically according to Misra and Fridovich [26] and expressed in units/gram of haemoglobin (U/g Hb). The relative activity of SOD was determined by means of vertical electrophoresis at 10% polyacrylamide gel electrophoresis (PAGE) and oxidation of nitro blue tetrazolium (NBT) following the Beauchamp and Fridovich method [27] (Hoeffer miniVE, Amersham, LKB, 2117, Bromma, Uppsala, Sweden). The band intensity was measured using TotalLab TL120, and results were expressed in arbitrary U/g Hb.

Catalase (CAT) activity in erythrocytes was assayed by means of the UV kinetic method at absorbance of 240 nm with the presence of H_2_O_2_ [28]. Activity was expressed as U/g Hb, calculated by using an extinction coefficient of 39.4 M^−1^ cm^−1^.

The level of malondialdehyde (MDA) in erythrocytes was quantified by measuring the formation of thiobarbituric acid reactive substances (TBARS) [29, 30]. The absorbance of the colored MDA-TBARS complex was measured at a wavelength of 535 nm and expressed in nM MDA/g Hb.

Nitrite concentration (NO_2_^−^) in plasma was determined with Griess reagent [31] on a microplate reader at 540 nm (plate reader, Mod. A1, Nubenco Enterprises, ICN). The results were expressed in *μ*M.

The determination of carbonyl groups (PCC) was performed spectrophotometrically with 2,4-dinitrophenylhydrazine at 365 nm [32]. The concentration of carbonyl groups was calculated on the basis of the absorption coefficient for this chromogen (*a* = 22.000 M^−1^ cm^−1^); the results are shown in *μ*M.

Alpha 1-acid glycoprotein (AGP) determined using Native PAGE, haptoglobin (Hp), and 3-nitrotyrosine (3-NT) levels in plasma of rats was determined using SDS-PAGE and Imunobloth methods with polyclonal antibody produced in rabbit (Sigma-Aldrich, St. Louise, USA) and monoclonal anti-3-nitrotyrosine antibody produced in mouse (Sigma-Aldrich, St. Louise, USA) [33, 34].

### 2.5. Determination of LDH

Total lactate dehydrogenase (LDH) was determined by following the initial rate of pyruvate reduction to lactate [34]. The activity of LDH was expressed in units per liter (U/L). Isoenzyme forms of LDH (LDH_1_-LDH_5_) were detected by PAGE technique (Hoeffer MiniVE, LKB, 2117, Bromma, Uppsala Sweden) using Tris-glycine buffer and sodium-lactate as substrates in the presence of nitro blue tetrazolium chloride [35]. Band intensity was measured densitometrically using TotalLab TL 120. The relative activity of isoenzymes was shown in percentages.

### 2.6. Native Gel Electrophoresis of Plasma Proteins

Vertical polyacrylamide gel electrophoresis at pH 8.6 (alkaline-PAGE) was carried out with Hoeffer miniVE cell (Amersham, LKB, 2117, Bromma, Uppsala Sweden) at 120 V and room temperature for 2 h. The gel used (0.75 mm thick) consisted of 4.5% T staking gel and 8% T separation gel (T% is an expression representing the concentration of acrylamide plus bisacrylamide in the gel). The electrode and migration buffers consisted of 0.19 M glycine and 0.024 M Tris at pH 8.6. After electrophoresis, proteins were stained using Coomassie blue 0.1% [36]. The band intensity was measured using TotalLab TL120. Results were shown in percentages in relation to the total area. The albumin : globulin ratio was calculated by dividing albumin content by the sum of *α*-, *β*-, and *γ*-globulins.

### 2.7. Micronucleus Test

The preparation and staining of bone marrow cells for the micronucleus test were performed according to Schmid [37] and Mavournin et al. [38]. After 24 hours of treatment, the bone marrow cells were flushed out with fetal calf serum, and the cells were suspended through centrifugation, smeared, and stained with May-Grünwald and Giemsa solution. A total of 1000 polychromatic erythrocytes were scored for each animal at a magnification of 100x (oil immersion) using a microscope (Leica, Germany). The PCE/NCE ratio was calculated to determine the cytotoxic effects of the adrenaline. All slides were coded and scored blind.

### 2.8. Statistical Analysis

Statistical significance of differences of all examined parameters was determined by means of the ANOVA test followed by Dunnett test. Normality tests were first performed for all groups using the d'Agostino-Pearson omnibus test. Data were expressed as means ± standard error (S.E.). Significance level was set at *P* < 0.05. Statistical analysis was done using GraphPad Prism 7.0 Software, CA, USA.

## 3. Results

**Figure 1:**
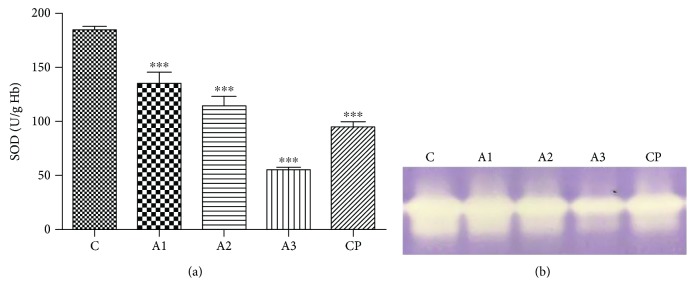
SOD activity after treatment with adrenaline (a), electrophoregram (b). Control group - C, groups treated with adrenaline doses (A1-0.75 mg/kg; A2-1.5 mg/kg; A3-3 mg/kg body weight); group treated with cyclophosphamide (CP). Data are expressed as means ± SE. ^∗∗∗^*P* < 0.001 vs. control group.

Results of the analysis of total activity of Cu, Zn-SOD enzyme in rats treated with adrenaline are shown in Figure 1. It was observed that adrenaline treatment significantly decreased (*P* < 0.001) the total enzyme activity by 26.09%, 38.09%, and 69.97%, respectively, in relation to the control group. Cyclophosphamide, as positive control, also reduces activity of SOD (48.54%, *P* < 0.001). The decreased activities of SOD biochemical assay were verified by the results of electrophoresis as shown in Figure 1(b).

**Figure 2:**
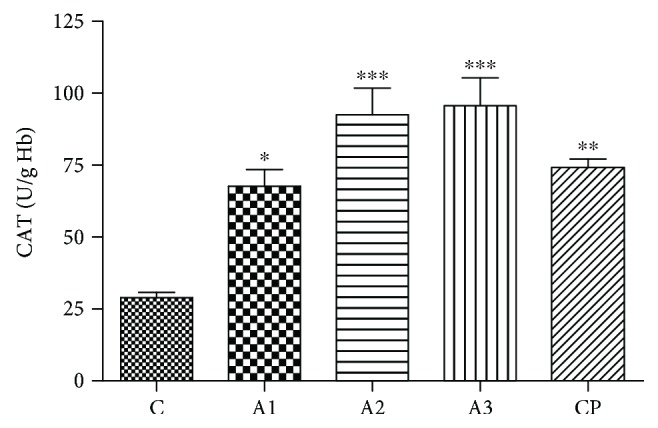
CAT activity after treatment with adrenaline. Control group - C, groups treated with adrenaline doses (A1-0.75 mg/kg; A2-1.5 mg/kg; A3-3 mg/kg body weight); groups treated with cyclophosphamide (CP). Data are expressed as means ± SE. ^∗^*P* < 0.05, ^∗∗^*P* < 0.01, ^∗∗∗^*P* < 0.001 vs. control group.

Significant increase in CAT activity was identified at all concentrations of adrenaline (Figure 2). The lowest concentration of adrenaline (0.75 mg/kg) induced significant increases (67.66 ± 5.83 U/g Hb, *P* < 0.05) in activity of CAT while the higher concentrations (1.5 mg/kg and 3 mg/kg) of adrenaline induce a more evident increase (92.50 ± 9.35 U/g Hb and 95.65 ± 9.73 U/g Hb, *P* < 0.001) compared to the control group (28.92 ± 1.83 U/g Hb). Similarly, the activity of CAT was significantly increased (*P* < 0.01) after treatment with cyclophosphamide.

**Figure 3:**
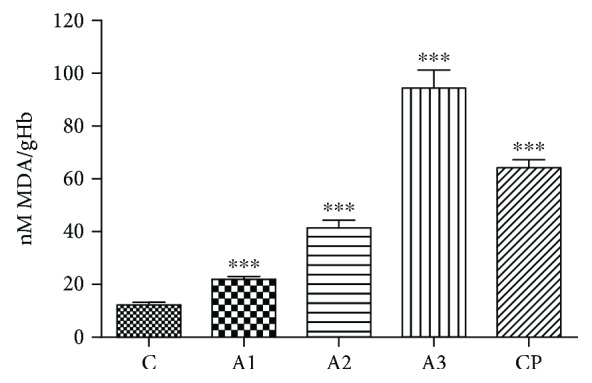
The level of MDA after treatment with adrenaline. Control group - C, groups treated with adrenaline doses (A1-0.75 mg/kg; A2-1.5 mg/kg; A3-3 mg/kg body weight); group treated with cyclophosphamide (CP). Data are expressed as means ± SE. ^∗∗∗^*P* < 0.001 vs. control group.

In Figure 3, MDA levels after treatment with adrenaline are shown. Adrenalin exerted a significant increase in a dose-dependent manner (1.84-, 3.41-, 7.78-, and 5.29-fold, *P* < 0.001) in MDA levels in rats compared to the untreated group (Figure 3). Also, cyclophosphamide induced a significant rise (*P* < 0.001) in MDA levels in respect to the control.

**Figure 4:**
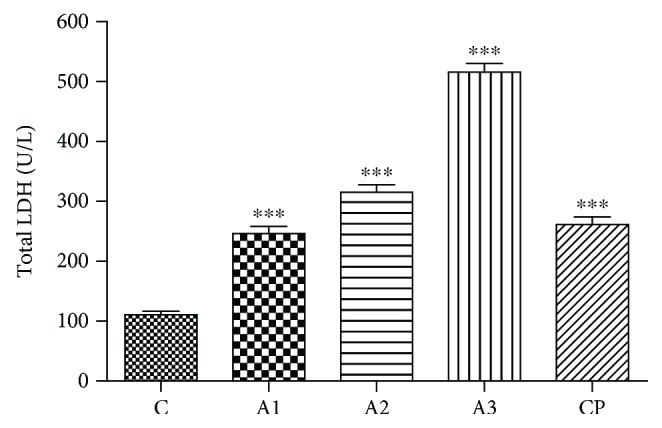
The total LDH level after treatment with adrenaline. Control group - C, groups treated with adrenaline doses (A1-0.75 mg/kg; A2-1.5 mg/kg; A3-3 mg/kg body weight); groups treated with cyclophosphamide (CP). Data are expressed as means ± SE. ^∗∗∗^*P* < 0.001 vs. control group.

**Figure 5:**
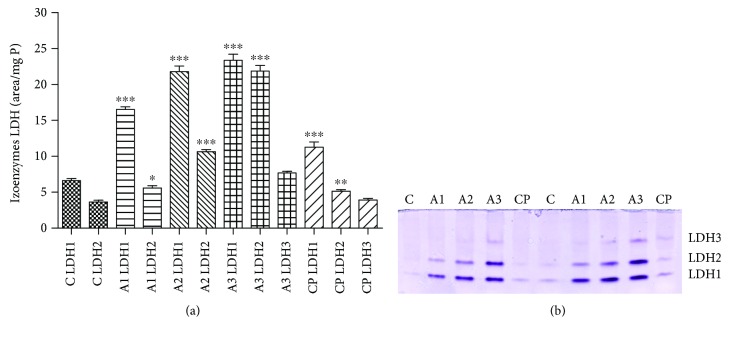
Relative distribution of LDH1-LDH5 isoenzymes (a) with electrophoretic profiles (b) after treatment with adrenaline. Control group - C, groups treated with adrenaline doses (A1-0.75 mg/kg; A2-1.5 mg/kg; A3-3 mg/kg body weight); group treated with cyclophosphamide (CP). Data are expressed as means ± SE. ^∗^*P* < 0.05, ^∗∗^*P* < 0.01, ^∗∗∗^*P* < 0.001 vs. control group.

Results of the total LDH and its relative isoenzyme distribution in rats treated with adrenaline are shown in Figures 4 and 5. There was clearly an increase (*P* < 0.001) in total activity of LDH in the group treated with all doses of adrenaline compared to the control group. Cyclophosphamide induces a less profound effect than the group treated with 3.5 mg/kg dose of adrenaline, but the total activity of LDH is also significantly increased (*P* < 0.001) in comparison to the control.

In Figure 5, it was observed that the LDH_1_ isoenzyme shows an evident increase (*P* < 0.001) in rats treated with adrenaline in respect to the control group. The lower but statistically significant effect (*P* < 0.05, *P* < 0.001) was evident in the activity of LDH_2_ isoenzyme after adrenaline treatment compared to the control group. Similarly, significant intensity bands for LDH_1_ and LDH_2_ isoenzymes were noticed in the group of rats treated with higher concentrations of adrenaline. The activity of isoenzymes LDH_1_ and LDH_2_ was also significantly increased (*P* < 0.01, *P* < 0.001) in rats treated with cyclophosphamide.

**Figure 6:**
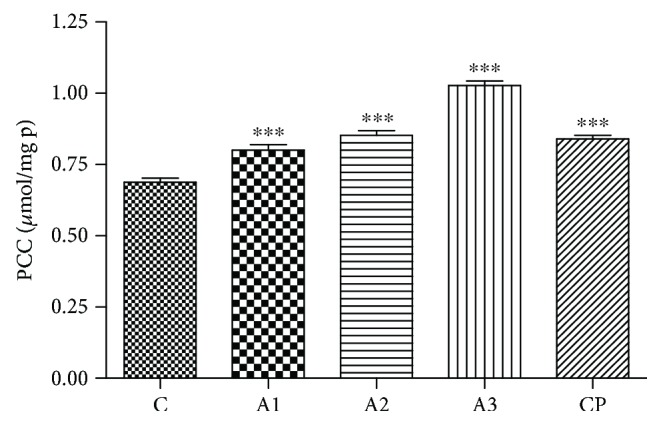
The PCC levels after treatment with adrenaline. Control group - C, groups treated with adrenaline doses (A1-0.75 mg/kg; A2-1.5 mg/kg; A3-3 mg/kg body weight); group treated with cyclophosphamide (CP). Data are expressed as means ± SE. ^∗∗∗^*P* < 0.001 vs. control group.

The effect of adrenaline on PCC levels in rats is presented in Figure 6. Compared to untreated rats, the significant elevation (*P* < 0.001) of PCC levels after treatment with all tested doses of adrenaline was noticed (Figure 6). Rats treated with cyclophosphamide also showed a significant rise (*P* < 0.001) in PCC levels in comparison to control.

**Figure 7:**
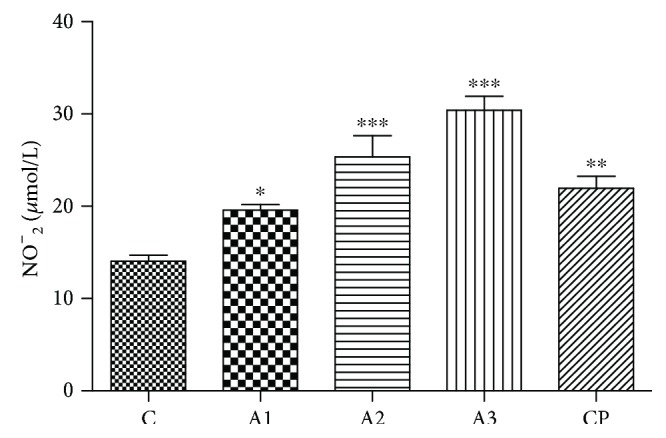
The level of NO2- in rats after treatment with adrenaline. Control group - C, groups treated with adrenaline doses (A1-0.75 mg/kg; A2-1.5 mg/kg; A3-3 mg/kg body weight); group treated with cyclophosphamide (CP). Data are expressed as means ± SE. ^∗^*P* < 0.05, ^∗∗^*P* < 0.01, ^∗∗∗^*P* < 0.001 vs. control group.

The level of NO_2_^−^ in rats after treatment with adrenaline is shown in Figure 7. Adrenaline induced a significant elevation of NO_2_^−^ level in a dose-dependent manner. The highest concentration (3 mg/kg) of adrenaline induced the most distinct increases of NO_2_^−^ (*P* < 0.001) compared to the control group. Positive control also induces a significant increment in the level of NO_2_^−^ (*P* < 0.01).

**Figure 8:**
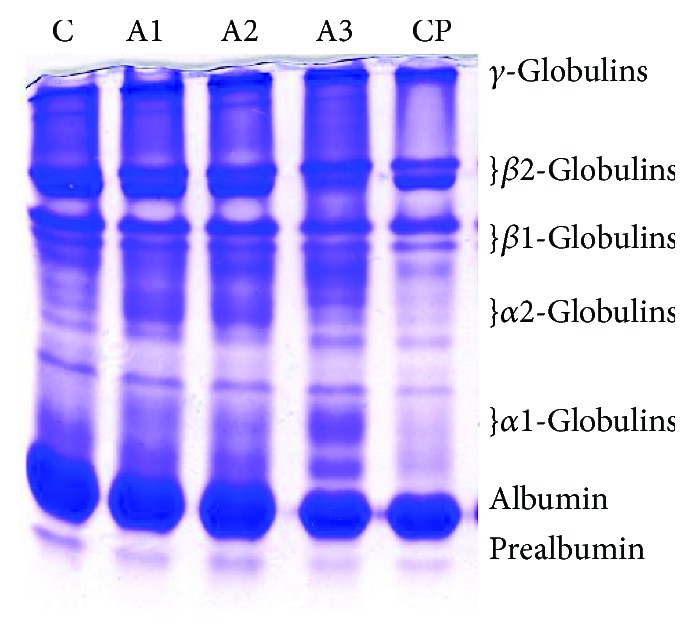
Representative electrophoregram of plasma proteins with Native PAGE after treatment with adrenaline. Control group- C, groups treated with adrenaline doses (A1-0.75 mg/kg; A2-1.5 mg/kg; A3-3 mg/kg body weight); groups treated with cyclophosphamide (CP).

The results of the electrophoretic distribution of plasma proteins are shown in Figure 8, while the results of their relative distribution are given in Table 1.

In our studies, reduced albumin concentration in adrenaline treatment has been demonstrated, and this decrease is dose-dependent. Adrenaline has been shown to reduce albumin concentration by 5.81%, 16.09%, and 18.75% depending on the dose level, while due to exposure to cyclophosphamide, the decrease of albumin is 9.62%. By comparing the results of albumin level in all groups with the control group, a statistically significant decrease in albumin was observed in the adrenaline-treated group with 1.5 and 3.0 mg/kg (*P* < 0.01). A significant increase in *α*1 fraction (AGP, *α*1-antitrypsin) versus the control group was observed in groups treated with adrenaline in doses of 0.75 mg/kg and 1.5 mg/kg (*P* < 0.01), and 3 mg/kg (*P* < 0.001), with increases by 42.97%, 45.09%, and 252.79%, respectably. The *α*2-fraction (Hp, ceruloplasmin, and *α*2-macroglobulin) was affected only by adrenaline treatment in doses of 0.75 mg/kg, 1.5 mg/kg, and 3.0 mg/kg and increases by 80.40% (*P* < 0.05), 150.67% (*P* < 0.01), and 79.36% (*P* < 0.05). A significant decrease (*P* < 0.05) of the *β*1-fraction (transferrin, haemopexin, and *β*-lipoproteins) versus the control group was noticed in groups treated with adrenaline in doses of 0.75 mg/kg (18.88%), 1.5 mg/kg (27.63%), and 3 mg/kg (19.32%). The *β*2-fraction (fibrinogen, C3, and *β*2-microglobulin) in groups treated with adrenaline was decreased by 27.78% (dose 0.75 mg/kg), 28.24% (1.5 mg/kg), and 50.69% (3 mg/kg), which was significantly lower (*P* < 0.001) than in the control group. In prealbumin and *γ*-globulin fractions, there were no significant differences between treated and control groups. A significant decrease (*P* < 0.05) in the ratio (A : G) versus control group was noticed in groups treated with adrenaline in doses of 1.5 mg/kg and 3 mg/kg.

**Figure 9:**
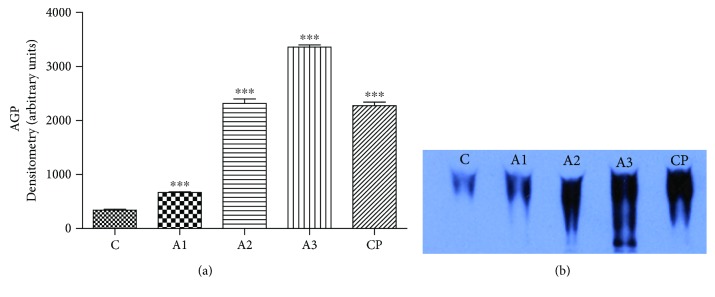
Immunohistochemical detection of APG on Native PAGE. The level of AGP (a) with electrophoregram (b) after treatment with adrenaline. Control group - C, groups treated with adrenaline doses (A1-0.75 mg/kg; A2-1.5 mg/kg; A3-3 mg/kg body weight); group treated with cyclophoshamide (CP). Data are expressed as means ± SE. ^∗∗^*P* < 0.01, ^∗∗∗^*P* < 0.001 vs. control group.

The significant increase (*P* < 0.001) in AGP levels was noticed in rats treated with adrenaline, especially at higher doses (1.5 mg/kg and 3 mg/kg) in respect to the control group (Figure 9). Related results of AGP levels on electropherogram were also detected.

**Figure 10:**
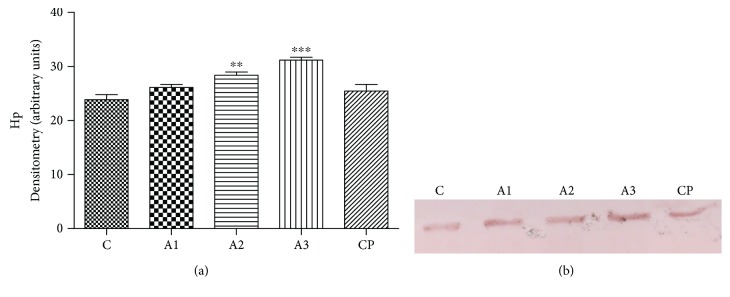
The level of haptoglobin (a) with electrophoregram (b) after treatment with adrenaline. Control group - C, groups treated with adrenaline doses (A1-0.75 mg/kg; A2-1.5 mg/kg; A3-3 mg/kg); group treated with cyclophosphamide (CP). Data are expressed as means ± SE. ^∗∗^*P* < 0.01, ^∗∗∗^*P* < 0.001 vs. control group.

The effect of adrenaline treatment on level of haptoglobin (Hp) in rats is presented in Figure 10. The significant difference (*P* < 0.01, *P* < 0.001) was observed in the level of Hp in rats treated with 0.75 mg/kg, 1.5 mg/kg, and 3 mg/kg of adrenaline, versus the control group, respectively. These results conformed with the electrophoretic profile.

**Figure 11:**
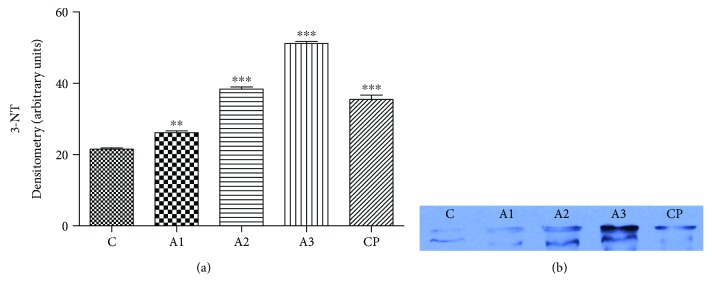
The level of nitrotyrosine (a) with electrophoregram (b) after treatment with adrenaline. Control group - C, groups treated with adrenaline doses (A1-0.75 mg/kg; A2-1.5 mg/kg; A3-3 mg/kg body weight); group treated with cyclophosphamide (CP). Data are expressed as means ± SE. ^∗∗^*P* < 0.01, ^∗∗∗^*P* < 0.001 vs. control group.

The effect of adrenaline treatment on level of 3-nitrotyrosine (3-NT) in rats is presented in Figure 11. A significant difference (*P* < 0.001) was evident in the level of nitrotyrosine in rats treated with all doses (1.5 mg/kg and 3 mg/kg) of adrenaline while a 0.75 mg/kg dose of adrenaline affects (*P* < 0.01) on the rise at the 3-NT level. In that manner, the intensity of the band was most evident at 3 mg/kg of adrenaline in the electrophoretic profile.

The results of the micronucleus (MN) test in bone marrow of rats treated with adrenaline are shown in Table 2. Adrenaline induced a significant increase (*P* < 0.001) in the frequency of micronucleated polychromatic erythrocytes (MNPCE) at higher concentrations (1.5 mg/kg, 3 mg/kg) when compared with the negative control group. Also, significant and dose-dependent decreases in polychromatic erythrocyte/normochromatic erythrocyte (PCE/NCE) ratio were seen in higher doses of adrenaline (1.5 mg/kg, 3 mg/kg). As expected, cyclophosphamide (60 mg/kg) induced a significant increase (*P* < 0.001) in MNPCE and decreases in the PCE/NCE ratio in respect to the control.

## 4. Discussion

In the last few decades, researchers focus on detecting harmful effects of chemical substances that are used as drugs in order to protect human health. In this study, we investigated toxic effects of adrenaline on Wistar rats using various parameters (SOD, CAT, MDA, NO_2_^−^, LDH, PCC, AGP, 3-NT, and APP) and obtained results unequivocally indicate that adrenaline at doses of 0.75 mg/kg, 1.5 mg/kg, and 3 mg/kg exerts oxidative and nitrative stress in rats.

In this study, SOD activity in erythrocytes was significantly decreased while CAT activity was significantly increased in rats treated with adrenaline compared to the untreated group. SOD and CAT are the most important antioxidant enzymes in the defense system against ROS [39]. These results may indicate that administration of adrenaline in rats caused disruption in oxidant/antioxidant balance. Superoxide anions generated in oxidative metabolism of adrenaline may stimulate oxidation of adrenaline and thus increase the amount of free radicals [11]. Decreased SOD activity in treated rats indicated that excess level of superoxide anions induced attenuation of SOD activity. On the other hand, increased CAT activity found in rats treated with adrenaline implies that CAT has an important protective role in removing free radicals produced in cells. This assumption is supported by studies of Djelić et al. [10] and Radakovic et al. [12] where catalase in cotreatment with adrenaline reduced DNA damage mediated by free radicals in human lymphocytes. Our results are in accordance with the study of Pereira et al. [40] who reported that adrenaline plays a role in the oxidative stress in the lymphoid organs since adrenodemedullation affected the activities of antioxidant enzymes.

We observed that the treatment with all doses of adrenaline clearly increased MDA levels in rat erythrocytes. Polyunsaturated fatty acids of the membrane are one of favored oxidation targets of ROS due to its oxygen-rich environment [41]. Our findings imply that adrenaline via free radicals induces enhancement in lipid peroxidation. This is in agreement with the study of Romana-Souza et al. [16] where treatment with a high level of adrenaline resulted in a significant increment of lipid peroxidation in four days in murine dermal fibroblasts. An *in vitro* study of Bukowska et al. [42] demonstrated increased lipid peroxidation in human peripheral blood mononuclear cells after treatment with catechol. Increased lipid peroxidation indicates damage on cell membranes caused by adrenaline. Analysis of total LDH activity in this investigation confirms disturbance of the plasma membrane integrity since increased LDH activity was found in rats treated with adrenaline. In order to determine the type of damaged tissue, LDH isoenzyme distribution was estimated. A higher level of LDH_1_ and LDH_2_ isoenzymes was found in the treated group which points on damage of cardiomyocytes of adrenaline. A similar effect was revealed in rats treated with cyclophosphamide. Our results suggest that cell membrane damage in cardiac muscle is responsible for an increase in LDH leakage which resulted as a consequence of oxidative stress in rats exposed to adrenaline [43]. There is evidence that cardiotoxicity may originate from adrenochrome, an oxidative product of adrenaline [4]. It was demonstrated that adrenochrome promotes redox cycling process with subsequent production of ROS [44]. This oxidative product of adrenaline has been detected in the heart, skeletal muscle, liver, and blood [9]. In this study, oxidative damage of proteins has been manifested as an increased level of carbonyl groups, in plasma of rats treated with adrenaline. As a consequence of oxidative damage to proteins, their functions as receptors, enzymes, or transport or structural proteins can be disturbed [45]. Consistent with our results, Romana-Souza et al. [16] reported increased levels of carbonylated proteins in skin fibroblast cultures after treatment with adrenaline. They indicate that adrenaline stimulates production of ROS/RNS and increases protein oxidative damage considering that the detrimental effect was abolished when cells were treated with inhibitors of free radicals. In addition, increased protein carboxylation was detected in human blood cells after treatment with catechol [42].

We observed that rats treated with all doses of adrenaline induced significant elevation of nitrite oxide (NO) which is manifested as a rise in the level of nitrite concentration (NO_2_^−^). Excessive NO synthesis often causes myocardium damage and loss of contractile function [46]. The toxicity of NO is reflected to its ability to react with superoxide to produce potent oxidant peroxynitrite (ONOO^−^) [47]. Therefore, this result points on nitrosative stress in rats treated with adrenaline. Namely, ONOO^−^ may spontaneously decompose to yield NO_2_^−^ and high reactive radical ^•^OH. Forming ONOO^−^ can take part of lipid peroxidation in reaction with unsaturated fatty acid-containing liposomes. In addition, ONOO^−^ may influence on protein participation in signal transduction mechanisms [48]. It was found that high concentrations of ONOO^−^ often lead to necrotic-type cell death [49]. Supportive evidence about the presence of nitrosative stress is revealed by our findings that an increase in the level of 3-NT was detected in the adrenaline-treated group. Consistent with our result is the study of Romana-Souza et al. [16] who reported an increased level of nitrotyrosine in mouse skin after treatment with a high level of adrenaline.

The mechanism of protein tyrosine nitration in biological systems has been well described [50]. Nitrotyrosine formation has been observed in various cardiovascular diseases [50–52]. An increased level of 3-NT may cause alternation of protein function, protein-protein interactions, and cell signaling [50, 53, 54]. Also, one of the consequences of adrenaline application is the acute phase response, which we demonstrated by evaluating APPs. One of the most important metabolic changes during the acute phase is the production of APPs which are released from the liver into the plasma [55]. This occurs within a few hours, and these proteins remain elevated as long as the inflammatory stimulus persists, making them perfect indicators of inflammation or injury, and useful for predicting the outcome of disease. Their only flaw is that they are poorly specific. APPs have been widely used in human and veterinary medicine as biomarkers of diseases, inflammatory processes, and various infections [56, 57]. Classification of APPs can be done according to the magnitude of the increase (positive APPs) or decrease (negative APPs) in their serum concentrations during the acute phase response [58]. Some of the APPs (*α*1-antitrypsin, and *α*2-macroglobulin) have antiprotease activity designed to inhibit phagocyte proteases and to minimize tissue damage. Others (*α*1-acid glycoprotein) have antibacterial or scavenging activity (haptoglobin, serum amyloid A, and C-reactive protein), by binding metabolites released from cellular degradation so they cannot be utilized by pathogen. Albumin, as a negative APP, is a major source of amino acids and is responsible for about 75% of the osmotic pressure of plasma. In this study, it has been shown that adrenaline reduced the albumin concentration by a dose-dependent level and also exposure to cyclophosphamide led to a decrease of albumin. Adrenaline causes an increased flux of free radicals, which can be due to the oxidation of thiol groups and the formation of albumin dimers or polymers, and consequently the reduction in osmotic pressure accurse.

The effect of the acute phase protein AGP in rats exposed to adrenaline was also estimated. A significant dose-dependent elevation in AGP following adrenalin treatment was observed. AGP is a positive APP with a normal concentration in the human plasma at 0.6–1.2 mg/mL [59]. The plasma concentration of AGP can increase from 2- to 10-fold when influenced by various factors, such as stress, inflammation drugs (phenobarbitone and rifampicin), burns, infections, and pregnancy. AGP possesses immunomodulatory activities and modulates neutrophil chemotactic migration and superoxide generation [60]. AGP significantly suppresses synthesis of IL-2, proliferation of lymphocytes, and platelet aggregation [61]. It has been shown that AGP protects neutrophil generation of ROS probably due to binding of free radicals [62]. AGP can bind to a number of metabolites such as heparin, histamine and serotonin, steroids, catecholamines, and pharmacological compounds. Increased AGP may affect pharmacokinetics by reducing the concentration of free drugs, by binding to the metabolically active fraction of the drug. Matsumoto et al. [63] reported that human AGP at physiological concentrations protects erythrocytes from H_2_O_2_. On the basis of the above-mentioned, we assume that the AGP level increases in response to oxidative stress after treatment with adrenaline. In this study, we detected an increased Hp level in rats treated with higher doses of adrenaline (1.5 and 3 mg/kg). Haptoglobin (Hp) is a positive acute-phase glycoprotein classified in *α*2 fraction together with fibrinogen and *α*-globulins with antiprotease activity [64]. Hp has a pronounced anti-inflammatory action, because of its ability to bind to heme of haemoglobin, forming an Hp-Hb complex. By forming this complex, Hp prevents the promotion of free radicals and its accumulation in endothelial cells which is catalysed by heme. Free Hb has the ability to catalyse the formation of hydroxyl radicals from H_2_O_2_. By binding to neutrophils, Hp may inhibit NADPH oxidase activation and therefore the production of reactive forms of oxygen associated with inflammation. The Hp-Hb complex, by removing free Hb, prevents renal injury that may occur when free Hb passes through the glomerular filter [65]. There is a great variability in antioxidant capacity, depending on Hp polymorphism, so individuals with Hp2-2 have a lower antioxidant capacity [65]. The Hp-Hb complex also binds nitric oxide or nitrogen monoxide (NO) [66]. This action is also phenotype-dependent. Due to its longer half-life, the Hp2-2/Hb complex scavenges more NO than the Hp1-1/Hb complex does [67, 68]. Haptoglobin can also modulate the immune response by binding to receptors on immune cells. Glucocorticoids and catecholamines activate haptoglobin synthesis whereas insulin exerts an opposite action. Since increased levels of extracellular Hb can occur due to impaired membrane integrity, we assumed that Hp scavenges free Hb as a result of lipid peroxidation. This fact coincides with our results of MDA analysis and leads to a conclusion that an elevated Hp level has a protective response to adrenaline-induced oxidative stress. Our findings unequivocally point that adrenaline induces an acute phase response. Although the APPs are a significant marker of inflammation and/or infection, it seems that these results give a new linkage between APPs and noninflammatory stress.

The possible genotoxicity of adrenaline was evaluated by a micronucleus test in bone marrow cells of rats. The results indicate that adrenaline expresses a genotoxic effect at higher concentrations (1.5 and 3 mg/kg) since it caused a significant induction of MN in the bone marrow of rats. This result is in compliance with the study of McGregor et al. [69] in which the adrenaline exhibited a mutagenic effect on mouse L5178Y lymphoma cells. In the Comet assay, adrenaline induced DNA damage in 3T3 cells of rats [6]. In our work, tested catecholamine (1.5 and 3 mg/kg) decreased the PCE/NCE ratio indicating its cytotoxicity in bone marrow. Muthuraman et al. [70] showed that adrenaline possessed a cytotoxic effect and affects DNA fragmentation in a dose-dependent manner in MDCK cells.

Several studies have implicated involvement of free radicals in the genotoxic action of adrenaline [12, 71]. Antioxidant enzymes CAT and SOD are the integral part of antioxidant defense mechanisms and have a considerable importance since they are involved in protection from free radicals. We assume that the antioxidants respond to oxidative stress caused by adrenaline since significant changes in the CAT and SOD activity in treated rats were detected. Martínez et al. [72] have classified catecholamines as potent oxidative mutagens. It has been suggested that catecholamine generates free radicals by autooxidation and redox cycling [44]. ROS have the ability to induce various types of DNA damage such as double-strand breaks (DSB), and this type of DNA damage is considered as a main contributor to MN induction [73]. We suggest that the increased induction of MN in the bone marrow of rats is a result of increased production of free radicals produced by oxidative metabolism of adrenaline. So, it should be expected that antioxidants could protect cells from toxic effects of adrenaline.

## 5. Conclusion

In summary, our results show that adrenaline induces oxidative and nitrative stress in Wistar rats, accompanied by changes in the activity of antioxidant enzymes, increased damage to lipids and proteins, increased level of NO_2_^−^ and nitrotyrosine derivate, damage of cardiomyocytes, and genotoxic damage. Also, adrenaline exerts acute-phase response through increased level of AGP and Hp and decreased level of serum albumin level. Therefore, obtained results may contribute to better understanding of adrenaline toxicity with aims at preventing its harmful effects.

## Figures and Tables

**Table 1:** Relative distribution of proteins with Native PAGE after treatment with adrenaline.

Groups	Intensity, % (mean ± SD)							Ratio (A : G)
	Prealbumin	Albumin	*α*1-	*α*2-	*β*1-	*β*2-	*γ*-Globulins	
*C*	2.66 ± 0.52	57.62 ± 3.80	5.19 ± 0.56	2.96 ± 1.62	15.89 ± 1.49	8.64 ± 0.79	7.04 ± 0.52	1.36 ± 0.22
A1	4.57 ± 0.57^∗∗^	54.32 ± 1.07	7.42 ± 1.03^∗∗^	5.34 ± 1.20^∗^	12.89 ± 0.70^∗^	6.24 ± 1.27^∗^	9.78 ± 1.38^∗^	1.20 ± 0.15
A2	3.66 ± 0.25	48.39 ± 3.72^∗∗^	8.53 ± 0.66^∗∗^	7.42 ± 1.36^∗∗^	11.50 ± 0.85^∗∗^	6.20 ± 1.28^∗^	9.43 ± 1.23	0.94 ± 0.22^∗^
A3	2.05 ± 0.32	46.86 ± 2.54^∗∗^	18.31 ± 3.01^∗∗∗^	5.31 ± 1.32^∗^	12.82 ± 1.52^∗^	4.26 ± 2.57^∗∗^	9.47 ± 1.56	0.88 ± 0.28^∗^
CP	3.69 ± 0.47^∗^	52.07 ± 1.25	6.07 ± 1.73	2.17 ± 1.52	16.14 ± 0.83	9.66 ± 0.84^∗∗^	9.11 ± 1.42	1.08 ± 0.23

^∗^ *P* < 0.05, ^∗∗^*P* < 0.01, and ^∗∗∗^*P* < 0.001. C: control group, groups treated with adrenaline doses (A1, 0.75 mg/kg; A2, 1.5 mg/kg; and A3, 3 mg/kg body weight); CP: group treated with cyclophosphamide.

**Table 2:** The frequency of micronuclei in bone marrow of Wistar rats treated with different doses of adrenaline.

Treatment	Treatment time (h)	Doses mg/kg	Total cell number	MNPCE (%)	PCE (%)
Negative control	24	0	5000	0.80 ± 0.37	49.24 ± 0.02

Adrenaline	24	0.75	5000	1.20 ± 0.49	47.92 ± 0.21
	24	1.5	5000	2.80 ± 0.37^∗^	46.40 ± 0.44^∗∗^
	24	3	5000	5.40 ± 0.24^∗^	43.43 ± 1.19^∗∗∗^

Cyclophosphamide	24	60	5000	14.40 ± 0.40^∗^	47.94 ± 0.38^∗∗∗^

Data are expressed as means ± SE. ^∗∗^*P* < 0.01 and ^∗∗∗^*P* < 0.001 vs. the control group.

## Data Availability

The data used to support the findings of this study are included within the article.
